# Breast MRI in nonpalpable breast lesions: a randomized trial with diagnostic and therapeutic outcome – MONET – study

**DOI:** 10.1186/1745-6215-8-40

**Published:** 2007-11-28

**Authors:** Nicky HGM Peters, Inne HM Borel Rinkes, Willem PTM Mali, Maurice AAJ van den Bosch, Remmert K Storm, Peter W Plaisier, Erwin de Boer, Adriaan J van Overbeeke, Petra HM Peeters

**Affiliations:** 1 Department of Radiology, University Medical Center Utrecht, Heidelberglaan 100, E01.132 3584 CX Utrecht, The Netherlands; 2 Department of Surgical Oncology, University Medical Center Utrecht, Heidelberglaan 100, G04.228 3584 CX Utrecht, The Netherlands; 3 Department of Radiology, Albert Schweitzer Ziekenhuis, van der Steenhovenplein 1 3300 AK Dordrecht, The Netherlands; 4 Department of Surgery, Albert Schweitzer Ziekenhuis, van der Steenhovenplein 1 3300 AK Dordrecht, The Netherlands; 5 Department of Radiology, Meander Medisch Centrum, lokatie Lichtenberg, Utrechtseweg 1603813 ES Amersfoort, The Netherlands; 6 Department of Surgery, Meander Medisch Centrum, lokatie Lichtenberg, Utrechtseweg 1603813 ES Amersfoort, The Netherlands; 7 Clinical epidemiology, Julius Center for Health Sciences and Primary Care, University Medical Center Utrecht, Heidelberglaan 100, STR 6.131 3584 CX Utrecht, The Netherlands

## Abstract

**Background:**

In recent years there has been an increasing interest in MRI as a non-invasive diagnostic modality for the work-up of suspicious breast lesions. The additional value of Breast MRI lies mainly in its capacity to detect multicentric and multifocal disease, to detect invasive components in ductal carcinoma in situ lesions and to depict the tumor in a 3-dimensional image. Breast MRI therefore has the potential to improve the diagnosis and provide better preoperative staging and possibly surgical care in patients with breast cancer. The aim of our study is to assess whether performing contrast enhanced Breast MRI can reduce the number of surgical procedures due to better preoperative staging and whether a subgroup of women with suspicious nonpalpable breast lesions can be identified in which the combination of mammography, ultrasound and state-of-the-art contrast-enhanced Breast MRI can provide a definite diagnosis.

**Methods/Design:**

The MONET – study (***M***R mammography ***O***f ***N***onpalpable Br***E***ast ***T***umors) is a randomized controlled trial with diagnostic and therapeutic endpoints. We aim to include 500 patients with nonpalpable suspicious breast lesions who are referred for biopsy. With this number of patients, the expected 12% reduction in surgical procedures due to more accurate preoperative staging with Breast MRI can be detected with a high power (90%). The secondary outcome is the positive and negative predictive value of contrast enhanced Breast MRI. If the predictive values are deemed sufficiently close to those for large core biopsy then the latter, invasive, procedure could possibly be avoided in some women. The rationale, study design and the baseline characteristics of the first 100 included patients are described.

**Trial registration:**

Study protocol number NCT00302120

## Background

Breast cancer is the most frequently occurring malignant disease in women with a lifetime risk of 1 in every 8 – 9 women [1]. Like many other Western countries, the Netherlands launched a screening program for breast cancer and since 1998, all women between 50 and 75 years of age are offered biannual mammographic examination. This program reveals approximately 4000 suspicious, nonpalpable breast lesions each year in the Netherlands [2]. In clinical practice, another 3000 nonpalpable lesions are detected annually in women with a high genetic risk or a history of breast cancer [3]. Of all suspicious nonpalpable breast lesions, 10–55% of lesions turn out to be malignant (ductal carcinoma in situ or invasive carcinoma) after large core needle biopsy (LCNB) depending on the aggressiveness of the referral of patients which varies across countries [4]. Subsequently, these patients are scheduled for surgical removal of the malignant breast tumor. In a relatively high proportion of these patients (approximately 20–30%), several surgical procedures (re-excisions or conversions from lumpectomy towards mastectomy) are needed to achieve complete removal of the primary tumor. The reasons for this include unanticipated invasiveness, multifocality, axillary lymph node involvement or incomplete removal of the primary tumor [5]. A more accurate preoperative diagnosis could help to reduce the number of surgical procedures and invasive biopsies.

In recent years there has been an increasing interest in MRI as a non-invasive diagnostic modality for further characterizing suspicious breast lesions detected with mammography or ultrasound. In a recent meta-analysis, we found that the sensitivity and specificity of MRI to diagnose breast cancer is 0.90 (95% CI 0.88 – 0.92) and 0.72 (95% CI 0.67 – 0.77) respectively [6]. Although these results are promising, the diagnostic performance is not sufficiently high to obviate LCNB for a definitive diagnosis. We believe that the additional value of MRI of the breast lies mainly in its capacity to detect multicentric, multifocal and bilateral disease [7-13], to detect invasive components in ductal carcinoma in situ (DCIS) lesions [8,11,13,14], to depict the tumor in a 3-dimensional image [8,11,14,15] and to depict breast cancer in dense breast tissue [15,16]. Breast MRI therefore has the potential to improve the diagnosis of invasive breast cancer and the preoperative staging of the breast. To assess this potential, we started a randomized clinical trials (the MONET – study: ***M***R mammography ***O***f ***N***onpalpable br***E***ast ***T***umors, clinical trials: study protocol number NCT00302120 [17]). The purpose of this trial is to assess whether performing contrast enhanced Breast MRI can reduce the number of invasive procedures in patients with nonpalpable suspicious breast lesions. Therefore, we will evaluate whether performing preoperative contrast-enhanced Breast MRI in women referred for surgical treatment, reduces the number of surgical procedures. Furthermore, we will assess whether a subgroup of women with suspicious nonpalpable breast lesions can be identified in which the combination of mammography, ultrasound and state-of-the-art contrast-enhanced Breast MRI can provide a definite diagnosis. If Breast MRI in addition to mammography and ultrasound will turn out to have a high positive predictive value, biopsies could possibly be prevented in some women in the future.

## Study design and methods

### Subjects

Women with nonpalpable suspicious breast lesions (BI-RADS category 3, 4, or 5) detected on mammography or breast ultrasound, who are referred for histological analysis of the lesion, are eligible for the study. Exclusion criteria are age below 18 or above 75 years, breast surgery or radiation therapy of the breast less than 9 months prior to inclusion, pregnancy or lactation, claustrophobia, severe obesity (> 130 kg), general contraindications for MRI (i.e. cardiac pacemaker, metal implants or history of severe allergic reaction after administration of contrast agent), inability to maintain in prone position for one hour, medically unstable patients and severe coagulopathies or use of anti-coagulants that cannot be discontinued. Written informed consent will be obtained from all patients. The study has been approved by the ethical boards of the participating hospitals.

### Study design

The MONET – study is a randomized controlled trial. Patients will be recruited in 3 hospitals: two large community teaching hospitals and one University Medical Center. In all participating centers, Breast MRI is not part of routine clinical practice in the work-up of patients with suspicious nonpalpable breast lesions. We aim to include a total of 500 patients with nonpalpable, mammographically suspicious breast lesions who are referred for biopsy. After giving informed consent, patients will be randomized in the control group or the MRI group. Patients in the control group (n = 250) will undergo routine medical care. This includes mammography, ultrasound, and the lesion is sampled by means of large core needle biopsy. In case of malignancy (DCIS or invasive carcinoma), the lesion is surgically removed. The MRI group (n = 250) will undergo routine clinical care and in addition, contrast enhanced Breast MRI in the University Medical Center prior to the large core needle biopsy. The MRI scan will be performed within one week after mammography and before LCNB which will be performed within one week after MRI to avoid a diagnostic delay.

### Randomization

Randomization is stratified by hospital. In each hospital, randomization within strata is blocked with a fixed block size. Randomization is performed by an independent trial center. If a patient meets the inclusion criteria and has provided informed consent, the physician contacts the trial center by phone. The trial center will perform the randomization of the patients.

### Time schedule

Patient recruitment will take place between 2006 and 2008. Between January and November 2006, each month approximately 15 patients were included in the MONET – study.

### Breast MRI

Breast MRI will be performed on a 3-T clinical MR system (Achieva, Philips, Best, The Netherlands). Patients will be placed in prone position. A dedicated phased-array bilateral breast coil (MRI devices, Würzburg, Germany) will be utilized for simultaneous imaging of both breasts. The scan protocol will include a transverse high-resolution T1-weighted fast gradient echo fat-suppressed series (TE/TR 1.7/4.5 msec; inversion delay SPAIR 130 msec; flip angle 10°; FOV 340 × 340 mm^2^, acquired voxel size 0.66 × 0.66 × 1.6 mm^3^, reconstructed voxel size 0.66 × 0.66 × 0.80 mm^3^) and a transverse T2-weighted fat suppressed spin echo series (TE/TR 120/9022 msec; inversion delay SPAIR 125 msec; flip angle 90°; FOV 340 × 340 mm^2^, acquired voxel size 1.01 × 1.31 × 2.0 mm^3^, reconstructed voxel size 0.66 × 0.66 × 2.00 mm^3^). Both series will be used to study the morphology of the lesion. A diffusion-weighted fat-suppressed series (TE/TR 61/5000 msec; inversion delay SPAIR 70 msec; flip angle 90 °; FOV 320 × 320 mm^2^; acquired voxel size 2.22 × 2.52 × 4.00 mm^3^, reconstructed voxel size 1.33 × 1.33 × 4.00 mm^3^; b-values 0, 150, 499 and 1500 seconds/mm^2^) will be acquired to assess the cellularity of the lesion. Finally, dynamic contrast-enhanced fat-suppressed T1-weighted gradient echo images (TE/TR 1.3/3.4 msec; flip angle 10°; FOV 320 × 320 mm^2^, acquired voxel size 0.91 × 0.91 × 2.00 mm^3^, reconstructed voxel size 0.83 × 0.83 × 1.00 mm^3^; dynamic scan duration 60 sec) will be acquired before and immediately after administration 0.1 mmol/kg Gadolinium-DTPA (Magnevist, Schering, Germany) to study the contrast enhancement of the lesions and herewith the perfusion of the lesion. All patients will receive this scan-package with a total scan duration of less than 30 minutes.

### Interpretation of Breast MRI

MR images of the breast will be interpreted on soft copy using a Picture Archiving and Communications System (PACS, Philips, Best, The Netherlands) that allows manual window level setting. MR images will be interpreted by two radiologists independently and in case of discrepancy, consensus will be sought. The radiologists will have access to the mammograms and ultrasound examination, but will be blinded for the results of the LCNB, following clinical practice. The MR images will be interpreted following the guidelines of the BI-RADS-MRI classification system proposed by the American College of Radiology [18]. Classification of the lesions will be based on lesion morphology, enhancement pattern and enhancement kinetics (persistent, plateau, or washout) [19,20]. Level of suspicion will be reported on a scale of 0 – 6: 0 additional imaging required; 1 normal; 2 benign; 3 probably benign, 6-month follow-up MRI recommended; 4 suspicious for malignancy; 5 highly suggestive of malignancy; 6 known malignancy.

### LCNB

LCNB will be performed under ultrasound guidance or stereotactically. A minimum of four 14 Gauge biopsy specimens per lesion is acquired. The histological diagnosis of the tissue sampled by means of the LCNB provides the definitive diagnosis. Based on histology, patient management will be planned: 'benign lesions' require mammographic follow-up, 'normal breast tissue' requires a repeat LCNB or open breast biopsy and in case of a 'high risk lesion' (i.e. lobular carcinoma in situ, atypical ductal or lobular hyperplasia), 'in-situ carcinoma' or 'invasive carcinoma' require image-guided needle localization followed by surgical excision with appropriate axillary staging (i.e. sentinel node biopsy or axillary lymph node dissection). Patient management will be the same for the MRI and the control group.

If in the intervention group an additional, mammographically occult lesion is detected on the MR images that requires tissue sampling, second-look ultrasound will be performed to determine whether the lesion is ultrasonographically evident and suitable for ultrasound-guided tissue sampling. If the lesion cannot be visualised by means of second-look ultrasound, MRI-guided large core needle biopsy will be performed.

### Surgery

Depending on the size of the tumor, the size of the breast and patient preferences, the lesion will be removed following the institutions' guidelines for breast cancer management. MR images will be shown to and discussed with the surgeon before the operation. The number and type of surgical procedures (lumpectomy, re-excision, mastectomy, sentinel node biopsy or axillary lymph node dissection) will be reported until 1 year after the LCNB.

### Primary outcome

To evaluate whether performing preoperative Breast MRI will improve breast cancer management, we will collect data on all biopsies (including biopsies performed for lesions that were detected on MRI only) and all surgical procedures during 1 year of follow-up after the LCNB. The number of biopsies and surgical procedures in patients in the MRI group will be compared to the number of procedures in the control group.

### Secondary outcome

Previous studies have shown that the positive predictive value of nonpalpable, BI-RADS 5 lesions detected on mammography is 96% [21]. If these lesions are classified as BI-RADS-MRI 5 as well, we expect the positive predictive value to increase further. In the 250 women who will undergo Breast MRI (intervention group), the positive and negative predictive values of Breast MRI in combination with mammography and ultrasound will be determined. If the predictive values are high (in certain subgroups, e.g patients with microcalcifications and dense breast lesions on mammography), LCNB could be omitted in the future in some women. Since all patients in the MONET – study will undergo LCNB, following clinical practice, the number of patients in whom LCNB could possibly be omitted in the future will be estimated based on the calculated predictive values of the MRI.

## Statistical considerations

### Sample size

The statistical power of the study was calculated for the primary endpoint (reduction in the number of surgical procedures). Based on data of previous studies on nonpalpable suspicious breast lesions and literature, we expect 23% of patients with suspicious nonpalpable breast lesions to require more than one surgical procedure to remove all tumorous tissue [19,22-26]. This number includes patients with mammographically occult multifocal and multicentric disease (8%), patients who require a surgical re-excision because all tumorous tissue could not be removed in one procedure (10%) and patients with a DCIS lesion with a mammographically occult invasive component in the surgical specimen (5%) [19,22-26]. We expect that additional contrast enhanced Breast MRI will reduce this number to 11% due to earlier detection of invasiveness, multifocality and muticentricity and a better 3D depiction of the tumor. With a statistical power of 90% to detect this 12% reduction as significant (p < 0.05, two-sided), we will require 250 women in the control group and 250 women in the MRI group (95% confidence interval 6% to 19%). We consider a reduction larger than 5% clinically relevant.

### Data-analysis

We will compare the number of all biopsies and surgical procedures within one year after the first LCNB between the MRI group and the control group. To compare the diagnostic performance of MRI (in combination with mammography and ultrasound) and LCNB, a two-by-two table will be constructed in which the results of breast MRI in addition to mammography and ultrasound will be compared to the results of the histology of the tissue sampled at the large core needle biopsy. The positive predictive value will be calculated by dividing the number of correctly identified positives by the total number of positive Breast MRI's. The negative predictive value will be calculated by dividing dividing the number of correctly identified negatives by the total number of negative MRI's. Analyses will be performed for specific subgroups, i.e. microcalcifications and dense breast lesions on mammography. If the positive predictive value of mammography, ultrasound and Breast MRI is sufficiently close the positive predictive value of large core needle biopsy in patients with nonpalpable breast lesions in all patients or in a subgroup of patients, the biopsy in these patients may be replaced by MRI in the future. However, whether MRI can indeed replace LCNB in clinical practice will have to be assessed in a separate study.

## Preliminary results

Between January 2006 and November 2006, 174 patients were eligible for the MONET – study. 108 patients were included in the study (recruitment rate 62%). The reasons for non-participation are listed in Table 1. Eight patients were excluded after randomization (no available histology (n = 5), unanticipated obesity (n = 1), technical failure of MR scanner (n = 1), age over 75 years (n = 1)). Three of the excluded patients were randomized in the control group and 5 in the MRI group. The characteristics of the 100 patients included so far in the MONET – study are summarized in Table 2. The mean age was 56.1 years (range 39.9 – 75.0 years). In these patients, a total of 109 lesions were detected: 108 lesions were detected on mammography and 1 lesion on ultrasound imaging. The majority of the lesions were microcalcifications and classified as BI-RADS 3 or 4. The histological diagnosis of the lesions included 2 non-representative, 9 normal breast tissue, 62 benign lesions, 21 non-invasive carcinomas and 19 invasive carcinomas. So, 35.4% of lesions turned out to be malignant (non-invasive and invasive) after LCNB. Forty-five patients were randomized into the MRI group and 55 into the control group.

**Table 1:** Reasons for non-participation

**Reason non-participation**	**Number of patients (%)**
Unable to lie in prone position	1 (1.5%)
Metal implants	6 (9.0%)
Severe obesity	1 (1.5%)
Claustrophobia	16 (24.0%)
Geographical distance from teaching hospital to MRI too long	18 (27.0%)
Clinical indication for MRI of the breast	1 (1.5%)
Personal reasons	23 (35.0%)
**Total**	**66**

**Table 2:** Characteristics of the first 100 patients included in the MONET – study

n	**100**
Referred from University Medical Center	**30**
Mean age (*range*)	**56.1 **(*39.9 – 75.0*)
Number of lesions detected on mammography	**108**
*density only*	*41*
*microcalcifications only*	*54*
*both*	*9*
*other*	*2*
*unknown*	*2*
BI-RADS classification mammography	
*3*	*45*
*4*	*47*
*5*	*11*
*unknown*	*5*
Number of lesions detected on ultrasound (*mammographically occult lesions*)	**1**
BI-RADS classification ultrasound	
*3*	*1*
*4*	*-*
*5*	*-*
*unknown*	*-*
Diagnosis LCNB	113*
*non-representative*	*2 (1.7%)*
*normal breast tissue*	*9 (8.0%)*
*benign*	*62 (54.9%)*
*non-invasive carcinoma*	*21 (18.6%)*
*invasive carcinoma*	*19 (16.8%)*

* 4 lesions were sampled twice because of inconclusive results

## Discussion

The purpose of the MONET – study is to evaluate whether performing contrast-enhanced Breast MRI in addition to mammography and/or ultrasound in patients with nonpalpable suspicious breast lesions will improve breast cancer management, i.e. to reduce the number of surgical procedures and/or the number of large core needle biopsies.

Many well conducted large clinical studies have been performed on the diagnostic performance of Breast MRI [5,6]. Only very few studies assessed the impact of Breast MRI on patient management. Two large observational studies have been performed to assess the value of Breast MRI for preoperative staging of patients with breast cancer [22,27]. The underlying assumption of both studies is that preoperative staging of the breast with MRI leads to the detection of mammographically occult multifocal and multicentric breast cancer, and that by removing these additional malignant lesions, the breast cancer recurrence rate could be reduced. Fischer et al compared the recurrent cancer rate in patients who did and did not undergo preoperative staging of the breast with MRI [22]. The reported recurrent cancer rate was indeed lower in the MRI group (1.2% (1/86)) than in the non-MRI group (6.5% (9/138)) after a mean follow-up time of 41 months. The percentage of contralateral tumors was lower in the MRI group as well: 1.7% (2/121) versus 4.0% (9/225). These differences were found to be statistically significant (p < 0.05) [22]. The recurrent cancer rate will also be assessed in the MONET – study: 5 years after completion of the MONET – study, the difference in recurrent cancer rate between patients from the MRI group and the control group will be assessed. Another study evaluated the change in surgical treatment after preoperative staging with MRI in patients with breast cancer [27]. Of the 267 patients that were scheduled for breast conserving therapy, the surgical plan of 69 patients (26%) was altered to more extensive surgery based on information obtained from preoperative staging with MRI. In 44 of these patients (64%) the alteration was considered to be appropriate based on pathological verification of malignancy in the surgical specimens [27]. Both authors advise preoperative staging with contrast-enhanced MRI in patients with breast cancer [22,27].

Randomized clinical trials assessing the therapeutic consequences of performing preoperative Breast MRI have not yet been performed. Moreover, the above mentioned studies included a mixture of patients with palpable and nonpalpable breast tumors. We believe that the nonpalpable lesions are the most challenging to remove in one attempt since these lesions cannot be seen or palpated at the end of the preoperatively inserted wire and the number of these nonpalpable lesions in increasing due to the widespread introduction of screening programs.

In conclusion, the aim of the MONET – study is to assess the diagnostic and therapeutic consequences of performing MRI of the breast in addition to mammography and ultrasound in patients with nonpalpable suspicious breast lesions.

## Abbreviations

MRI Magnetic Resonance Imaging

MONET ***M***R mammography ***O***f ***N***onpalpable Br***E***ast ***T***umors

LCNB Large Core Needle Biopsy

95% CI 95% Confidence Interval

DCIS Ductal Carcinoma in Situ

BI-RADS Breast Imaging Reporting and Data System

kg Kilograms

T Tesla

TE Echo Time

TR Repetition Time

msec milliseconds

SPAIR Spectral Presaturation Attenuated by Inversion Recovery

FOV Field-of-View

mm millimetres

mmol millimols

DTPA Gadopentetate Dimeglumine

PACS Picture Archiving and Communications System

3D 3-dimensional

## Competing interests

The author(s) declare that they have no competing interests.

## Authors' contributions

NP drafting manuscript, participated in design and coordination of the study IB, WM, MB, RS, PPl, EB, AO participated in design and coordination of the study PPe assisted in drafting manuscript, participated in design and coordination of the study All authors revised the manuscript and approved the final version of the manuscript.

## MONET – study group

University Medical Center Utrecht, the Netherlands

Department of Radiology: W.P.Th.M. Mali, G. Stapper, A.M. Fernandez – Gallardo, M.A.A.J. van den Bosch, I. van den Berk, W. Veldhuis, A. Hamersma, C. Haaring, C. Meeuwis, N.H.G.M. Peters. Department of Surgery: S. van Esser, I.H.M. Borel Rinkes, R. van Hillegersberg. Department of Pathology: P.J. van Diest. Department of Clinical Epidemiology: P.H.M. Peeters.

Albert Schweitzer Hospital Dordrecht, the Netherlands

Department of Radiology: R. Storm, P. van der Valk, A. ter Braak, R. Roozendaal. Department of Surgery: P.W. Plaisier, R.J. Oostenbroek. Department of Pathology: T.M. Teune, R.J. Heinhuis, P.J. Westenend.

Meander Medisch Centrum Amersfoort, the Netherlands

Department of Radiology: R. Gruyters, E. de Boer, J. van der Pol. Department of Surgery: A. van Overbeeke.

## References

[B1] Jemal A, Siegel R, Ward E, Murray T, Xu J, Smigal C, Thun MJ (2006). Cancer statistics, 2006. CA Cancer J Clin.

[B2] (2003). Incidence of breast cancer in the Netherlands 1999/2000. Eleventh report of the Netherlands cancer registry..

[B3] (2002). National evaluation of breast cancer screening in the Netherlands.

[B4] Smith-Bindman R, Chu PW, Miglioretti DL, Sickles EA, Blanks R, Ballard-Barbash R, Bobo JK, Lee NC, Wallis MG, Patnick J, Kerlikowske K (2003). Comparison of screening mammography in the United States and the United kingdom. JAMA.

[B5] Hrung JM, Sonnad SS, Schwartz JS, Langlotz CP (1999). Accuracy of MR imaging in the work-up of suspicious breast lesions: a diagnostic meta-analysis. Acad Radiol.

[B6] Peters NH, Borel Rinkes IH, Zuithoff NP, Mali WP, Moons KG, Peeters PH (2008). Meta-analysis of MR imaging in the diagnosis of breast lesions. Radiology.

[B7] Boetes C, Mus RD, Holland R, Barentsz JO, Strijk SP, Wobbes T, Hendriks JH, Ruys SH (1995). Breast tumors: comparative accuracy of MR imaging relative to mammography and US for demonstrating extent. Radiology.

[B8] Hlawatsch A, Teifke A, Schmidt M, Thelen M (2002). Preoperative assessment of breast cancer: sonography versus MR imaging. AJR Am J Roentgenol.

[B9] Kacl GM, Liu P, Debatin JF, Garzoli E, Caduff RF, Krestin GP (1998). Detection of breast cancer with conventional mammography and contrast-enhanced MR imaging. Eur Radiol.

[B10] Malur S, Wurdinger S, Moritz A, Michels W, Schneider A (2001). Comparison of written reports of mammography, sonography and magnetic resonance mammography for preoperative evaluation of breast lesions, with special emphasis on magnetic resonance mammography. Breast Cancer Res.

[B11] Mumtaz H, Hall-Craggs MA, Davidson T, Walmsley K, Thurell W, Kissin MW, Taylor I (1997). Staging of symptomatic primary breast cancer with MR imaging. AJR Am J Roentgenol.

[B12] Sardanelli F, Giuseppetti GM, Panizza P, Bazzocchi M, Fausto A, Simonetti G, Lattanzio V, Del MA (2004). Sensitivity of MRI versus mammography for detecting foci of multifocal, multicentric breast cancer in Fatty and dense breasts using the whole-breast pathologic examination as a gold standard. AJR Am J Roentgenol.

[B13] Schelfout K, Van GM, Kersschot E, Colpaert C, Schelfhout AM, Leyman P, Verslegers I, Biltjes I, Van Den HJ, Gillardin JP, Tjalma W, Van Der Auwera JC, Buytaert P, De SA (2004). Contrast-enhanced MR imaging of breast lesions and effect on treatment. Eur J Surg Oncol.

[B14] Hwang ES, Kinkel K, Esserman LJ, Lu Y, Weidner N, Hylton NM (2003). Magnetic resonance imaging in patients diagnosed with ductal carcinoma-in-situ: value in the diagnosis of residual disease, occult invasion, and multicentricity. Ann Surg Oncol.

[B15] Kristoffersen WM, Aspelin P, Sylvan M, Bone B (2003). Comparison of lesion size estimated by dynamic MR imaging, mammography and histopathology in breast neoplasms. Eur Radiol.

[B16] Amano G, Ohuchi N, Ishibashi T, Ishida T, Amari M, Satomi S (2000). Correlation of three-dimensional magnetic resonance imaging with precise histopathological map concerning carcinoma extension in the breast. Breast Cancer Res Treat.

[B17] (2006). www.clinicaltrails.gov. http://www.clinicaltrials.gov/ct2/show/NCT00302120?term=monet&rank=1.

[B18] Ikeda DM, Hylton NM, Kinkel K, Hochman MG, Kuhl CK, Kaiser WA, Weinreb JC, Smazal SF, Degani H, Viehweg P, Barclay J, Schnall MD (2001). Development, standardization, and testing of a lexicon for reporting contrast-enhanced breast magnetic resonance imaging studies. J Magn Reson Imaging.

[B19] Fischer U, Kopka L, Grabbe E (1999). Breast carcinoma: effect of preoperative contrast-enhanced MR imaging on the therapeutic approach. Radiology.

[B20] Kuhl CK (2000). MRI of breast tumors. Eur Radiol.

[B21] Hoorntje LE, Peeters PH, Mali WP, Borel R (2004). Is stereotactic large-core needle biopsy beneficial prior to surgical treatment in BI-RADS 5 lesions?
1498. Breast Cancer Res Treat.

[B22] Fischer U, Zachariae O, Baum F, von HD, Funke M, Liersch T (2004). The influence of preoperative MRI of the breasts on recurrence rate in patients with breast cancer
1497. Eur Radiol.

[B23] Hoorntje LE (2003). Nonpalpable breast lesions: challenges in diagnosis and treatment. Thesis.

[B24] Intra M, Zurrida S, Maffini F, Sonzogni A, Trifiro G, Gennari R, Arnone P, Bassani G, Opazo A, Paganelli G, Viale G, Veronesi U (2003). Sentinel lymph node metastasis in microinvasive breast cancer
1496. Ann Surg Oncol.

[B25] Verkooijen HM (2000). Stereotactic large-core needle biopsy for nonpalpable breast disease. The COBRA study.. Thesis.

[B26] Estourgie SH (2004). Excisional biopsy of breast lesions. Changes in the drainage pattern: a reproducibility study of lymphoscintigraphy. Thesis,.

[B27] Bedrosian I, Mick R, Orel SG, Schnall M, Reynolds C, Spitz FR, Callans LS, Buzby GP, Rosato EF, Fraker DL, Czerniecki BJ (2003). Changes in the surgical management of patients with breast carcinoma based on preoperative magnetic resonance imaging. Cancer.

